# Delineation of the genetic and clinical spectrum of Phelan-McDermid syndrome caused by *SHANK3* point mutations

**DOI:** 10.1186/s13229-018-0205-9

**Published:** 2018-04-27

**Authors:** Silvia De Rubeis, Paige M. Siper, Allison Durkin, Jordana Weissman, François Muratet, Danielle Halpern, Maria del Pilar Trelles, Yitzchak Frank, Reymundo Lozano, A. Ting Wang, J. Lloyd Holder, Catalina Betancur, Joseph D. Buxbaum, Alexander Kolevzon

**Affiliations:** 1 Seaver Autism Center, Icahn School of Medicine at Mount Sinai, New York, NY 10029 USA; 2 Department of Psychiatry, Icahn School of Medicine at Mount Sinai, New York, NY 10029 USA; 3 Department of Neurology, Icahn School of Medicine at Mount Sinai, New York, NY 10029 USA; 4 Department of Pediatrics, Icahn School of Medicine at Mount Sinai, New York, NY 10029 USA; 5 Department of Genetics and Genomic Sciences, Icahn School of Medicine at Mount Sinai, New York, NY 10029 USA; 6 Division of Neurology and Developmental Neuroscience, Department of Pediatrics, Baylor College of Medicine and Texas Children’s Hospital, Houston, TX 77030 USA; 7 Sorbonne Université, INSERM, CNRS, Neuroscience Paris Seine, Institut de Biologie Paris Seine, 75005 Paris, France; 8 Department of Neuroscience, Icahn School of Medicine at Mount Sinai, New York, NY 10029 USA; 9 Friedman Brain Institute, Icahn School of Medicine at Mount Sinai, New York, NY 10029 USA; 10 Mindich Child Health and Development Institute, Icahn School of Medicine at Mount Sinai, New York, NY 10029 USA

**Keywords:** *SHANK3*, Phelan-McDermid syndrome, 22q13 deletion syndrome, Sequence variants, Phenotype, Autism spectrum disorder, Intellectual disability

## Abstract

**Background:**

Phelan-McDermid syndrome (PMS) is a neurodevelopmental disorder characterized by psychiatric and neurological features. Most reported cases are caused by 22q13.3 deletions, leading to *SHANK3* haploinsufficiency, but also usually encompassing many other genes. While the number of point mutations identified in *SHANK3* has increased in recent years due to large-scale sequencing studies, systematic studies describing the phenotype of individuals harboring such mutations are lacking.

**Methods:**

We provide detailed clinical and genetic data on 17 individuals carrying mutations in *SHANK3*. We also review 60 previously reported patients with pathogenic or likely pathogenic *SHANK3* variants, often lacking detailed phenotypic information.

**Results:**

*SHANK3* mutations in our cohort and in previously reported cases were distributed throughout the protein; the majority were truncating and all were compatible with de novo inheritance. Despite substantial allelic heterogeneity, four variants were recurrent (p.Leu1142Valfs*153, p.Ala1227Glyfs*69, p.Arg1255Leufs*25, and c.2265+1G>A), suggesting that these are hotspots for de novo mutations. All individuals studied had intellectual disability, and autism spectrum disorder was prevalent (73%). Severe speech deficits were common, but in contrast to individuals with 22q13.3 deletions, the majority developed single words, including 41% with at least phrase speech. Other common findings were consistent with reports among individuals with 22q13.3 deletions, including hypotonia, motor skill deficits, regression, seizures, brain abnormalities, mild dysmorphic features, and feeding and gastrointestinal problems.

**Conclusions:**

Haploinsufficiency of *SHANK3* resulting from point mutations is sufficient to cause a broad range of features associated with PMS. Our findings expand the molecular and phenotypic spectrum of PMS caused by *SHANK3* point mutations and suggest that, in general, speech impairment and motor deficits are more severe in the case of deletions. In contrast, renal abnormalities associated with 22q13.3 deletions do not appear to be related to the loss of *SHANK3*.

**Electronic supplementary material:**

The online version of this article (10.1186/s13229-018-0205-9) contains supplementary material, which is available to authorized users.

## Background

Phelan-McDermid syndrome (PMS, OMIM 606232) is a rare neurodevelopmental disorder characterized by neonatal hypotonia, global developmental delay, intellectual disability (ID), severely delayed or absent speech, and frequent autism spectrum disorder (ASD) [1]. The neurobehavioral phenotype of PMS is usually severe. In a prospective study of 32 PMS individuals, 77% manifested severe-to-profound ID and 84% met criteria for ASD using gold standard diagnostic tools [2]. Dysmorphic features are usually mild and include long eyelashes, large or prominent ears, bulbous nose, pointed chin, fleshy hands, and dysplastic toenails [1]. Additional features include gastrointestinal problems, seizures, motor deficits, structural brain abnormalities, renal malformations, lymphedema, and recurrent infections [1].

The major neurodevelopmental features of PMS are caused by deletions or mutations of the *SHANK3* gene, which encodes a scaffolding protein of the postsynaptic density of glutamatergic synapses. Most reported cases of PMS are caused by 22q13.3 deletions, which usually encompass many genes and can extend up to 9.2 Mb [2–4]. Genotype-phenotype analyses indicate that the size of the deletion and the number and/or severity of clinical manifestations are positively correlated [2, 4–7]. Specifically, correlations have been reported between deletion size and hypotonia [5–7], developmental delay [5–7], dysmorphic features [2, 7], speech abilities [4], social communication deficits related to ASD [2], and other medical conditions [2]. Furthermore, individuals with small terminal deletions may have more favorable developmental trajectories than those with larger deletions [8].

De novo truncating and missense mutations in *SHANK3* have been identified in cohorts ascertained for ASD [9–16] or ID [17–21]. In addition, there is a single report of two families ascertained for schizophrenia with mutations in *SHANK3*; affected individuals also had ID [22]. Despite the increasing number of mutations in *SHANK3*, their prevalence in PMS and more broadly in ASD is underestimated because clinical sequencing is still uncommon compared to chromosomal microarray. In addition, *SHANK3* has been poorly covered by whole exome sequencing due to high GC content [13, 23], and there is little in the PMS phenotype that would prompt a clinician to specifically target *SHANK3* for optimized Sanger sequencing. We and others estimate that *SHANK3* haploinsufficiency might account for up to 1% of more severely affected ASD cases [13, 23].

Given the dearth of identified cases with *SHANK3* mutations, analyses of PMS cohorts have largely focused on individuals with 22q13.3 deletions [2–8, 24]. Only two studies on PMS have included a few individuals carrying *SHANK3* mutations [2, 24]. These observations have been complemented by the description of a small number of individuals identified through *SHANK3* targeted sequencing in ASD cohorts [9–13]. Large-scale sequencing studies have been instrumental in revealing additional *SHANK3* mutations but have not provided detailed phenotypic information [14–16, 19–21].

The limited number of subjects with *SHANK3* mutations examined thus far, and the lack of systematic clinical evaluation have hindered the characterization of the phenotypic spectrum associated with *SHANK3* mutations. Here, we aimed to delineate the genetic spectrum of *SHANK3* mutations and their associated phenotype in relationship to PMS features.

## Methods

### Participants

**Fig. 1:**
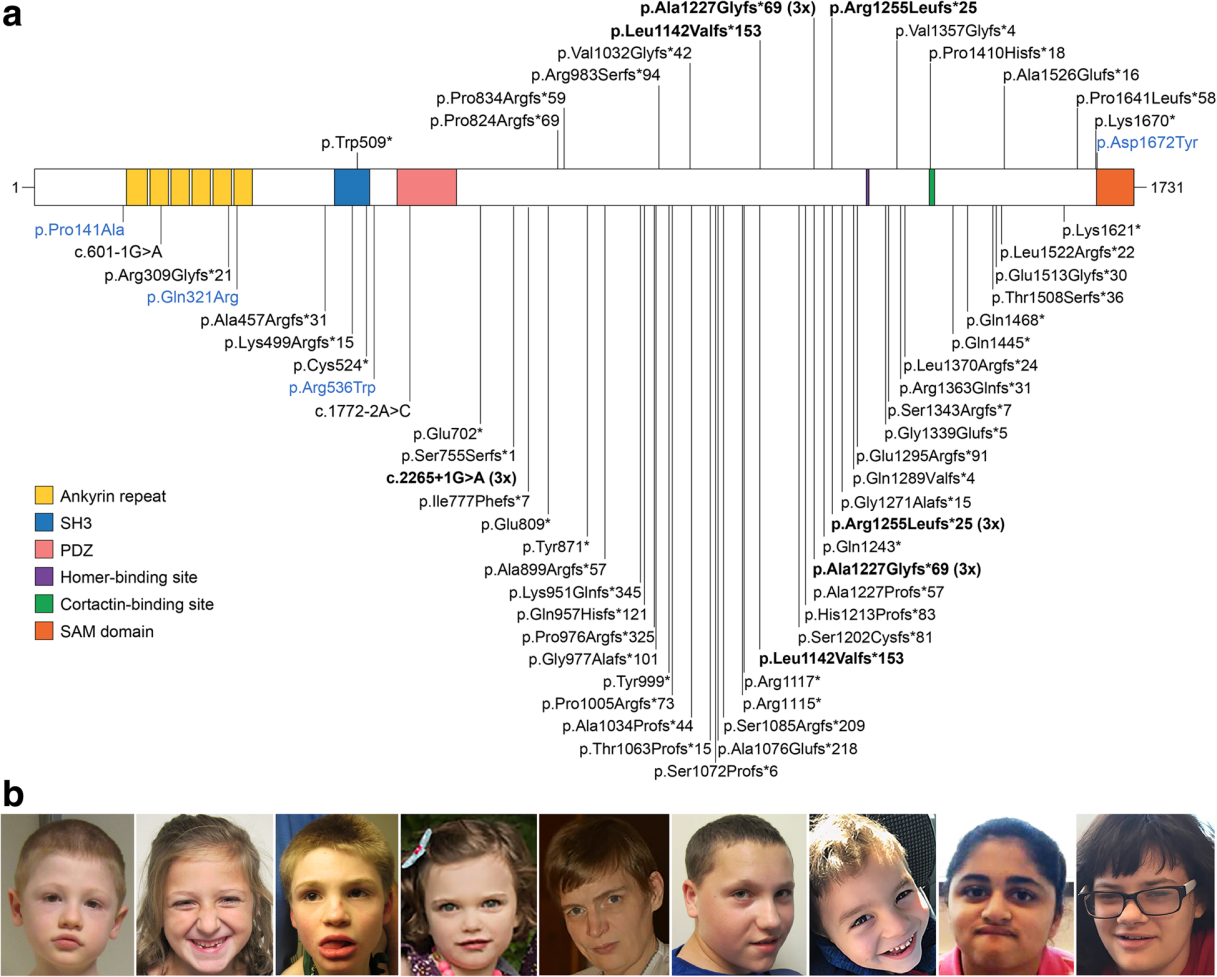
**a** Pathogenic and likely pathogenic *SHANK3* mutations. The mutations described in this study are shown in the upper panel and those reported in the literature or in ClinVar in the lower panel. Loss-of-function mutations are indicated in black and missense mutations in blue. Recurrent mutations are indicated in bold. Protein domains are from UniProt; the homer and cortactin binding sites are indicated as previously reported [9]. **b** Photographs of individuals with mutations in *SHANK3*, showing mild dysmorphic features

The study includes 14 participants (S1–S14) enrolled at the Seaver Autism Center for Research and Treatment at the Icahn School of Medicine at Mount Sinai, and three individuals (B1–B3) evaluated at Baylor College of Medicine. Individuals were referred through the Phelan-McDermid Syndrome Foundation, ongoing research studies, and communication between families. The study was approved by the Program for the Protection of Human Subjects at the Icahn School of Medicine at Mount Sinai and the Baylor College of Medicine Institutional Review Board. Parents or legal guardians provided informed consent for participation and publication. Consent was also obtained to publish the photos shown in Fig. 1.

### Genetic testing

All mutations were identified and/or validated by Clinical Laboratory Improvement Amendments (CLIA)-certified laboratories. The mutation in individual S1 was identified by whole exome sequencing (WES) and then validated by Sanger sequencing at the Seaver Autism Center [2] and by GeneDx. The mutation in S2 was identified by panel sequencing at the Michigan Medical Genetics Laboratories. The mutation in S3 was identified and validated at Seaver as previously reported [2] and further confirmed by Athena Diagnostics. The mutation in S4 was identified through clinical WES by the Columbia University Laboratory of Personalized Medicine. The mutations in S5, S11, and B1 were identified through clinical WES by the Medical Genetics Laboratory at the Baylor College of Medicine. The mutations in S6, S7, S9, S10, S12, and S14 were identified through clinical WES by GeneDx. The mutation in S8 was identified through clinical WES by AmbryGenetics. The variants in S13 were identified at the Seaver Autism Center and confirmed by GeneDx. The mutation in B2 and B3 was identified through clinical WES by Transgenomic.

Variants were described according to the Human Genome Variation Society guidelines. As reported previously [2], the human genome reference assembly (GRCh37/hg19 and GRCh38/hg38) is missing the beginning of exon 11 (NM_033517.1:c.1305_1346, 5′-cccgagcgggcccggcggccccggccccgcgcccggccccgg-3′, coding for 436-PSGPGGPGPAPGPG-449). We numbered nucleotide and amino acid positions according to the *SHANK3* RefSeq mRNA (NM_033517.1) and protein (NP_277052.1) sequence, in which this mistake has been corrected. Variants were interpreted according to the American College of Medical Genetics and Genomics (ACMG) guidelines [25].

### Review of previously reported *SHANK3* mutations

We searched the literature for pathogenic or likely pathogenic mutations in *SHANK3* and retrieved the molecular and clinical information (Additional file 1: Tables S1–S3). We also included mutations reported in ClinVar (http://www.ncbi.nlm.nih.gov/clinvar/). To avoid duplicate counting of affected individuals, we reviewed all available information (including gender, country of origin, and phenotype) and contacted the authors when doubts persisted. Individuals reported more than once are indicated in Additional file 1: Table S1.

### Clinical evaluation

Prospective clinical and psychological characterization was completed for 12 individuals seen at the Seaver Autism Center (S1–S4, S6–S8, S10–S14), including three previously reported (S1 and S3 [2] and S13 [26]). A battery of standardized assessments was used to examine ASD, intellectual functioning, adaptive behavior, language, motor skills, and sensory processing (see below). The medical evaluation included psychiatric, neurological, and clinical genetics examinations and medical record review. The evaluation of the individuals seen at Baylor College of Medicine (B1–B3) included parent interview, neurological examination, and medical record review. Their seizure phenotype and brain magnetic resonance imaging (MRI) findings were reported previously [24]. Two additional individuals (S5 and S9) received genetic testing through the Seaver Autism Center but were not evaluated clinically. Their caregivers completed surveys to capture developmental, medical, and behavioral health issues and were interviewed by phone.

#### ASD phenotype

Gold-standard ASD diagnostic testing included the Autism Diagnostic Observation Schedule, Second Edition (ADOS-2) [27], the Autism Diagnostic Interview-Revised (ADI-R) [28], and a clinical evaluation to assess Diagnostic and Statistical Manual for Mental Disorders, Fifth Edition (DSM-5) criteria for ASD [29]. The ADOS-2 and ADI-R were administered and scored by research reliable raters, and the psychiatric evaluation was completed by a board-certified child and adolescent psychiatrist. The ADOS-2 is a semi-structured observational assessment that provides scores in the domains of social affect, restricted and repetitive behavior, and a total score. A comparison score ranging from 1 to 10, with higher scores reflecting a greater number of symptoms, was calculated to examine symptom severity within each ADOS-2 domain and in total [30]. Nine individuals (S1–S4, S6, S8, S11, S13, S14) received module 1 of the ADOS, for children who are nonverbal or communicate using single words. Two individuals (S7, S10) received module 3, for children who are verbally fluent. The ADI-R is a structured caregiver interview that assesses ASD symptomatology within the domains of socialization, communication, and repetitive and restricted interests and behavior. A consensus diagnosis was determined for each participant based on results from the ADOS-2, ADI-R, and clinical evaluation using DSM-5.

#### Intellectual functioning

Global cognitive ability was measured using the Mullen Scales of Early Learning [31] (*n* = 10), the Stanford Binet Intelligence Scales, Fifth Edition [32] (*n* = 1), and the Differential Ability Scales, Second Edition (DAS-II) [33] (*n* = 1), depending on age and verbal ability. The Mullen is validated for children from birth to 68 months but is commonly used for older individuals with ID [34]. Developmental quotients were calculated using age equivalents divided by chronological age as has been done in previous studies [35]. For example, a nonverbal developmental quotient was computed by dividing the mean age equivalents on the visual reception and fine motor scales by the child’s chronological age and then multiplying by 100. The DAS-II is a measure of cognitive functioning that assesses a child’s verbal reasoning, nonverbal reasoning, and spatial abilities. A general conceptual ability can be calculated to assess overall intellectual functioning. The Stanford-Binet Intelligence Scales, Fifth Edition is an intelligence test that produces a nonverbal intellectual quotient (IQ), verbal IQ, and full scale IQ based on performance across five scales: fluid reasoning, knowledge, quantitative reasoning, visual-spatial, and working memory.

#### Adaptive behavior

The Vineland Adaptive Behavior Scales, Second Edition, Survey Interview Form (Vineland-II) [36] is a clinician-administered interview that assesses adaptive behavior in the domains of communication, daily living, socialization, and motor skills. The Vineland-II was completed for 11 individuals. The motor domain is intended for children ages 6 years and under but was assessed in all individuals given significant motor delays in this population. The Vineland-II was also used in conjunction with cognitive testing to identify the presence and severity of ID.

#### Language skills

Language milestones were assessed during the ADI-R (*n* = 11) and the psychiatric evaluation. Current expressive and receptive language abilities were assessed using the Mullen (*n* = 10), Vineland-II (*n* = 11), MacArthur-Bates Communicative Development Inventories [37] (*n* = 10), Peabody Picture Vocabulary Test, Fourth Edition [38] (*n* = 3), and Expressive Vocabulary Test [39] (*n* = 2).

#### Motor skills

Motor milestones were assessed during the ADI-R (*n* = 11) and the psychiatric evaluation (*n* = 12). Current motor skills were assessed using the Vineland-II (*n* = 11) and Mullen (*n* = 10) fine and gross motor skills domains. The Beery Visual-Motor Integration Test, 6th Edition [40] was completed when appropriate (*n* = 2).

#### Sensory processing

Sensory processing was assessed using the Short Sensory Profile [41] and the Sensory Assessment for Neurodevelopmental Disorders (SAND) [42]. The Short Sensory Profile is a 38-item caregiver report form that investigates daily life sensory experiences. The SAND is a standardized assessment that includes a clinician-administered observation and a 36-item corresponding caregiver interview. The scoring algorithm measures sensory hyperreactivity, hyporeactivity, and seeking behavior across visual, tactile, and auditory domains.

## Results

### *SHANK3* mutations

We report 17 individuals (including two monozygotic twins) with *SHANK3* mutations identified through WES or panel sequencing. The variants were distributed throughout the protein and included 13 frameshift, two nonsense, and one missense mutation (Table 1, Fig. 1a). Notably, we observed an identical frameshift mutation, c.3679dupG (p.Ala1227Glyfs*69), in three unrelated individuals. Mutations were confirmed to be de novo in 15 individuals and non-paternal or non-maternal in two (no DNA was available from the other two parents). In addition to a nonsense mutation, individual S13 carries a missense variant (p.Ser1291Leu) absent in the mother but present in the unaffected sister and in four individuals in the Genome Aggregation Database (gnomAD), suggesting it is likely benign, despite being predicted as damaging by several in silico tools (Additional file 1: Table S3). All other mutations are absent from the Exome Variant Server (EVS) and gnomAD. The missense mutation in S14 (p.Asp1672Tyr) affects a highly conserved residue and is predicted to be damaging by all algorithms used, including Polyphen-2, SIFT, PANTHER, MutPred2, Condel2, CADD, and M-CAP (Additional file 1: Table S3).

**Table 1:** *SHANK3* point mutations in 17 individuals described in this study

ID	Coding DNA change^a^	Protein change^b^	Genomic change (hg19)	Location	Effect	Inheritance	Variant classification [25]
S1^c^	c.1527G>A	p.Trp509*	chr22:g.51137146G>A	Exon 12	Nonsense	De novo	Pathogenic
S2	c.2471delC	p.Pro824Argfs*69	chr22:g.51158732delC	Exon 21	Frameshift	De novo	Pathogenic
S3^d^	c.2499delG	p.Pro834Argfs*59	chr22:g.51158760delG	Exon 21	Frameshift	De novo	Pathogenic
S4	c.2946_2949delCCGC	p.Arg983Serfs*94	chr22:g.51159207_51159210delCCGC	Exon 21	Frameshift	De novo	Pathogenic
S5	c.3095_3107delTGGGGGCCATCGA	p.Val1032Glyfs*42	chr22:g.51159356_51159368delTGGGGGCCATCGA	Exon 21	Frameshift	De novo	Pathogenic
S6	c.3424_3425delCT	p.Leu1142Valfs*153	chr22:g.51159685_51159686delCT	Exon 21	Frameshift	De novo	Pathogenic
S7	c.3679dupG	p.Ala1227Glyfs*69	chr22:g.51159940dupG	Exon 21	Frameshift	Non-paternal	Pathogenic
S8	c.3679dupG	p.Ala1227Glyfs*69	chr22:g.51159940dupG	Exon 21	Frameshift	De novo	Pathogenic
B1^e^	c.3679dupG	p.Ala1227Glyfs*69	chr22:g.51159940dupG	Exon 21	Frameshift	De novo	Pathogenic
S9	c.3764_3776delGGGCCCAGCCCCC	p.Arg1255Leufs*25	chr22:g.51160025_51160037delGGGCCCAGCCCCC	Exon 21	Frameshift	De novo	Pathogenic
B2, B3^e,f^	c.4065_4066delTG	p.Val1357Glyfs*4	chr22:g.51160326_51160327delTG	Exon 21	Frameshift	De novo	Pathogenic
S10	c.4229delC	p.Pro1410Hisfs*18	chr22:g.51160490delC	Exon 21	Frameshift	De novo	Pathogenic
S11	c.4577_4578delCC	p.Ala1526Glufs*16	chr22:g.51160838_51160839delCC	Exon 22	Frameshift	De novo	Pathogenic
S12	c.4906_4921dupTCCCCCTCGCCGTCGC	p.Pro1641Leufs*58	chr22:g.51169450_51169465dupTCCCCCTCGCCGTCGC	Exon 22	Frameshift	De novo	Pathogenic
S13^g^	c.5008A>T	p.Lys1670*	chr22:g.51169552A>T	Exon 22	Nonsense	Non-maternal	Likely pathogenic
	c.3872C>T	p.Ser1291Leu	chr22:g.51160133C>T	Exon 21	Missense	Non-maternal	Likely benign
S14	c.5014G>T	p.Asp1672Tyr	chr22:g.51169558G>T	Exon 22	Missense	De novo	Likely pathogenic

^a^NM_033517.1

^b^NP_277052.1 (Q9BYB0-1)

^c^S1 also has a de novo pathogenic 17q12 microduplication [62]. Reported previously [2, 14, 26]

^d^Reported previously [2, 26]

^e^Reported previously [24]

^f^Monozygotic twins

^g^Reported previously [26]. This individual has two variants in *SHANK3*; the missense variant is likely benign and is not shown in Fig. 1a

We also searched the literature and ClinVar for *SHANK3* mutations and assessed their pathogenicity. Variants listed in Additional file 1: Table S1 meet the following criteria: (1) loss-of-function variants (frameshift, nonsense, and splice site), or de novo missense variants predicted to be deleterious by several bioinformatics predictors, and (2) absent from control databases (EVS and gnomAD). After removing cases ascertained or reported multiple times, we identified 60 additional individuals from 55 families with *SHANK3* mutations classified as pathogenic or likely pathogenic according to ACMG [25]. All the mutations with parental samples available were de novo. Three families had multiple affected siblings, consistent with germline mosaicism [9, 22, 43]. Four de novo missense variants reported in children with ASD, ID, or infantile spasms (p.Thr337Ser, p.Ser1197Gly, p.Ala1214Pro, and p.Arg1255Gly) [15, 44–46] were classified as variants of uncertain significance because, although not present in controls, in silico predictions did not provide consistent evidence for pathogenicity (Additional file 1: Tables S1, S3). Given that *SHANK3* is highly constrained against missense variation (Exome Aggregation Consortium Z score 4.92) [47], further studies are needed to determine the pathogenicity of these and other missense variants.

Three of the mutations in our cohort are recurrent, having been previously observed in unrelated individuals (Fig. 1a, Additional file 1: Table S1). The mutation in S6, p.Leu1142Valfs*153, was reported in a boy with ASD [13]. The mutation c.3679dupG (p.Ala1227Glyfs*69), shared by three of our patients (S7, S8, B1), is within a stretch of eight guanines and has been identified in three independent cases [9, 15, 20]. p.Arg1255Leufs*25, present in S9, has been reported in three unrelated patients [13, 21]. The donor splice site at position c.2265+1 is another hotspot: there are three individuals with a G>A substitution [16, 24, 48], and one with a deletion of the same G (c.2265+1delG), shown to result in a frameshift (p.Ser755Serfs*1) [11]. Overall, there were four recurrent and 56 private pathogenic/likely pathogenic mutations in *SHANK3* (Fig. 1a, Additional file 1: Table S1).

We also searched for potentially deleterious variants inherited from unaffected parents or present in population controls (Additional file 1: Table S4). An inherited frameshift variant reported as pathogenic in two unrelated children with ASD [12, 49], and classified as damaging in the Human Gene Mutation Database, is in fact intronic when annotated in the correct reference sequence, NM_033517.1 [49], and is present 173 times in gnomAD (chr22:g.51135705dupG, hg19). An inherited substitution in a splice region (c.1772-4G>A) reported in ASD [12] is present seven times in gnomAD and is thus unlikely to be deleterious. gnomAD contains 21 variants predicted to be loss-of-function when annotated in the Ensembl canonical transcript ENST00000262795 (which is missing the beginning of exon 11 and contains three extra, unvalidated exons). When annotated in NM_033517.1, many of these variants are in fact intronic. The remaining 10 loss-of-function variants are all singletons; seven are flagged because they were found in sites covered in a limited number of individuals, which may indicate low-quality sites, one is located at the extreme 3′ end, and one has an abnormal allele balance. These findings confirm that truncating variants in *SHANK3* are highly penetrant and unlikely to be present in unaffected individuals.

Four in-frame deletions [10, 13, 19, 50] and one in-frame insertion [50] in *SHANK3* have been reported in ASD/ID (Additional file 1: Table S4). Three of these variants were inherited [10, 13, 50], and one was found in two controls [50], suggesting that some short in-frame deletions or insertions may be tolerated. An in-frame deletion of five amino acids (p.Gly1453_Ala1457del) reported in an ASD proband and his unaffected mother [10] was detected in six individuals in the gnomAD database. gnomAD lists 15 in-frame deletions or insertions (after annotation in NM_033517.1); six are on multiallelic sites, and four others are flagged because of low coverage. Among the remaining in-frame variants, p.Glu1230del was observed in five individuals and p.Gly1518del in four (Additional file 1: Table S4). These findings indicate that at least some in-frame variants in *SHANK3* can be present in seemingly unaffected individuals.

### Clinical phenotype of *SHANK3* haploinsufficiency

#### Phenotypic spectrum in the individuals from our cohort

Detailed clinical information of the 17 individuals (9 males and 8 females, 3–42 years old at evaluation) is summarized in Tables 2 and 3 and Additional file 2: Table S5.

**Table 2:** Main clinical features of individuals with *SHANK3* mutations

	S1	S2	S3	S4	S5^a^	S6	S7	S8	S9^a^	S10	S11	S12	S13	S14	B1	B2^b^	B3^b^	Total (%)
Gender	M	M	M	F	F	M	F	M	M	M	F	F	M	M	F	F	F	9 M, 8 F
Gestational age (wks)	36	39	40	Term	38	Term	33	40	39	Term	36.5	Term	40	41	40	36	36	
Birth weight (g)	2863	3400	3657	4000	3175	2948	1940	4111	4630	3230	3000	3728	2700	3090	2438	2551	2523	
Birth length (cm)	47	55	NK	53	NK	50	46	58	55	50	53	NK	48	51	48	NK	NK	
Postnatal growth																		
Age at examination (y)	12	5	7	3	9	5	7	9	12	9	6	42	15	4	14	14	14	3–42
Height (cm, percentile)	131 (< 1)	110 (27)	108 (< 1)	103 (93)	NK	109 (50)	125 (45)	148 (95)	142 (17)	135 (56)	111 (4)	170 (85)	149 (< 1)	97 (4)	151 (25–50)	145 (< 1)	145 (< 1)	5/16 (31%) short
Weight (kg, percentile)	32.6 (11)	20 (53)	18.8 (5)	17.2 (88)	NK	17.3 (50)	35 (96)	36 (78)	29.5 (3)	29.5 (55)	18.2 (7)	69.4 (82)	48.1 (18)	15.4 (19)	55.1 (75)	59.1 (75)	40.5 (12)	
OFC (cm, percentile)	56 (95)	50.8 (36)	52.7 (70)	50 (75)	NK	NK	51.2 (40)	54 (82)	NK	53 (64)	50.5 (27)	57 (99)	54.5 (40)	46 (< 1)	52.7 (30)	57 (98)	57 (98)	3/14 (21%) macrocephaly; 1 (7%) microcephaly
Psychomotor development																		
Sat independently (mo)	12	5–6	9–10	6	12	9	8–11	6	8–9	6	Normal	8	NK	5	8	6	6	
Walked independently (mo)	24	16	14	15	16	14	18	14	14–15	12	24	20	14	13	14	19	19	
First words and current language ability	3 y; now non-verbal	No speech; vowel sounds and sounds of pleasure present	No speech; previously had 10 signs but regressed to 3–4, babbling present, apraxic	No speech; uses about 5 signs with word approximations	Non-verbal	36 mo; 2-word phrases at 3.5 y; primarily single words with some phrase speech, stereotyped speech and echolalia	14 mo; first phrases at 24 mo, verbally fluent, complex speech	15 mo; never developed 2-word phrases, eventually lost all words	Non-verbal	8 mo; 2-word phrases by 24 mo, now verbally fluent	4 y; had approximately 10 words; currently uses no words	15 mo; phrase speech at 3 y; was verbally fluent until 12–13 y but currently uses no words	10–15 words by 18 mo; understands roughly 40 signs; comprehension and expressive language is limited	10 mo; uses a few word approximations, some signs, apraxic	At 3 y had approximately 200 words but only used 50 routinely; can speak in 2–3 word sentences but mostly echolalia	19 mo; combined words at 3.5 y; currently speaks in full sentences but developed word finding difficulties	19 mo; combined words at 3.5 y; spoke in full sentences but regressed at 9 y to only say 2–3 words, regained some vocabulary but fluctuating language	Currently non-verbal 9/17 (53%), fluent speech 3/17 (18%)
Intellectual disability (IQ or DQ)	Profound ID (Mullen: DQ 6.7, NVDQ 10.3, VDQ 3.1)	Profound ID (Mullen: DQ 21.3, NVDQ 30.1, VDQ 12.5)	Profound ID (Mullen: DQ 14.7, NVDQ 18.8, VDQ 10.6)	Profound ID (Mullen: DQ 16.5, NVDQ 19.5, VDQ 13.4)	ID (no testing available)	Severe ID (Mullen: CSS < 49, DQ 30.4, NVDQ 35, VDQ 25.8)	Mild ID (DAS-II: GCA 50, Verbal 52, NV reasoning 74, spatial 32, special NV 49)	Profound ID (Mullen: DQ 11.5, NVDQ 14.5, VDQ 8.5)	Severe ID (no testing available)	Mild ID (Stanford Binet: FSIQ 56, NVIQ 60, VIQ 56)	Profound ID (Mullen: DQ 10.5, NVDQ 13.2, VDQ 7.9)	Profound ID (Mullen: DQ 0.63, NVDQ 0.97, VDQ 0.29)	Profound ID (Mullen: DQ 10.4, NVDQ 15.5, VDQ 5.2)	Severe ID (Mullen: CSS < 49, DQ 26.4, NVDQ 33, VDQ 19.8)	Mild ID (BDI at 4 y: adaptive SS 65, cognitive 65, communication 65, fine motor 72, gross motor 69, social 65)	Mild ID (no testing available)	Mild ID (no testing available)	17/17 (100%)
Feeding difficulties	+ (chewing problems)	−	+ (regurgitation, oral motor dysfunction, difficulty consuming solid foods, PEG tube)	+ (oral motor dysfunction since birth, dysphagia, drooling, overeating, chewing problems)	+ (failure to thrive, g-tube)	−	− (drooling)	+ (dysphagia, drooling, may induce vomiting when over-eats)	+ (difficulty chewing, dysphagia)	+ (history of oral motor dysfunction, drooling)	+ (history of dysphagia)	+ (drooling, dysphagia)	+ (difficulty latching, currently gagging and choking behaviors, dysphagia, drooling)	+ (oral motor dysfunction)	−	+ (difficulty latching)	+ (difficulty latching)	13/17 (76%)
Hypotonia	+	+	+	+	+	+	+	+	+	+	+	+	+	+	−	+	+	16/17 (94%)
Gait abnormalities	+ (apraxic, hypotonic, toe-walking)	+ (toe-walking, unsteady, needs assistance)	+	+	+ (slow pace)	+ (toe walking)	+ (mildly hypotonic)	+	+	+	+ (apraxia)	+ (slow, hesitant and apraxic; previously reported as wide-based gait)	+ (mild but went through 6-month period in early childhood when he was unable to ambulate due to muscle weakness)	+	−	−	−	14/17 (82%)
Behavioral abnormalities																		
ASD	+	+	−	+	+	+	−	+	+	−	+	NK	+	+	+	−	−	11/16 (69%)
Hyperactivity	+	+	+	+	+	+	+	−	+	+	+	−	−	−	+	−	−	11/17 (65%)
Aggression	−	−	+	−	−	−	+	−	−	+	+	+	+	−	−	+	+	8/17 (47%)
Self-injury	−	−	+	−	−	−	−	+	−	−	−	+	−	−	−	NK	NK	3/15 (20%)
Sleep disturbance	−	+	+	+	+	−	−	−	+	−	+	+	+	+	+	−	−	10/17 (59%)
Pica	+	+	+	+	+	+	−	+	+	−	−	−	−	−	−	−	−	8/17 (47%)
Repetitive behaviors (type)	+ (stereotypic motor movements in upper and lower extremities, forced exhalations)	+ (spinning, hand-flapping, teeth grinding)	+ (repetitive motor mannerisms, stereotypic vocalizations)	+ (bouncing, tapping, upper extremity motor stereotypies)	+ (chewing, teeth grinding, breath holding)	+ (chewing, teeth grinding, hand flapping, stereotypic vocalizations)	+ (self-stimulation, insistence on routines)	+ (hand-flapping, chewing)	+ (hand-flapping)	+ (restricted interests, perseveration)	+ (teeth grinding, repeatedly taps objects, walks in circles)	+ (pacing, upper extremity motor stereotypies)	+ (hand flapping, chewing, stereotypic vocalizations, teeth grinding)	+ (chewing, hand flapping, repetitive jumping, stereotypic vocalizations)	+ (finger and toe tapping)	−	+ (chewing, tapping teeth with finger)	16/17 (94%)
Psychosis	−	−	−	−	−	−	−	−	−	−	−	+ (12–13 y)	−	−	−	−	−	1/17 (6%)
Regression (age and details)	+ (5 y; some language loss)	−	+ (6 y; motor regression and lost some sign language, at one point stopped walking for 6 weeks, slowly regained ambulatory skills; at 5 y, some language loss)	+ (15 mo; stopped babbling, loses motor skills when sick)	−	−	−	+ (3.5 y; language and motor skills, stopped walking, socially withdrawn and less responsive)	+ (3–4 y; loss of fine motor skills)	−	+ (4.5 y; loss of language and motor skills, lost ability to ambulate and eye contact, lethargic, developed unusual motor stereotypies, regression coincided with diagnosis of parasitic infection)	+ (12–13 y; intermittent periods of behavioral, motor, and language regressions sometimes preceded by viral infection, included psychiatric symptoms. Currently non-verbal and unable to walk unsupported)	+ (2 y; lost all words, 7 y; regression in handwriting [can no longer hold a pen], motor regression began roughly around when seizures started)	+ (12–18 mo; loss of babbling, loss of few words, eye contact, and gesturing to request)	−	+ (13 y; “manic-like” behavior)	+ (9–10 y; “manic-like” behavior)	11/17 (65%)
Neurological findings																		
Brain MRI (age)	Diffuse ventricular enlargement, colpocephaly, communicating hydrocephalus, thinning of parieto-occipital white matter and corpus callosum (8 y)	No MRI	Leukodystrophy (5 y)	Grossly normal but scattered areas of subtle FLAIR hyperintensity (2 y)	NK	Grossly normal but hyper-intensity in the left inferior parietal subcortical white matter possibly related to gliosis (4 y)	Normal (5 y)	Normal (3 y)	Normal (5, 9, and 11 y)	Normal (7 y)	Normal (4.5 y)	Normal (14 and 18 y)	Venous angioma (6 y); normal (16 y)	Normal (3 y)	Bilateral T2 hyper-intensities of posterior centrum semiovale (12 y)	Mild cerebellar tonsillar ectopia (14 y)	Normal (14 y)	Abnormal in 5/15 (33%)
Seizures (age of onset, type)	−	−	+ (5 y, Landau-Kleffner variant; 6 y epileptic encephalopathy)	−	−	−	−	−	− (10 y, suspected complex partial seizures)	−	−	−	+ (3 y febrile; 6 y focal; 15 y began 1–10 absence or partial seizures daily)	+ (4 y, generalized myoclonic seizures)	−	+ (14 y, atypical absence)	+ (7 y, atypical absence and tonic)	5/17 (29%)
Abnormal EEG	−	−	+ (localized sleep potentiated epileptiform discharges mainly in the midline and central regions during slow wave sleep)	+ (increased theta wave activity; bilateral K-complexes, and spindles and vertex waves during sleep. Left frontal spike and wave activity)	+ (spikes)	+ (spike and wave activity in frontotemporal lobes; no seizures)	−	−	+ (right frontal lobe spikes, slowing)	−	+ (left frontal spikes/polyspikes, intermittent polymorphic slowing [L>R] in the temporal region, and background slowing during sleep)	−	+ (high-voltage spike and sharp activity in frontal regions)	+ (occasional generalized polyspikes or polyspike-wave with shifting hemispheric predominance during sleep)	−	+ (no occipital dominant rhythm)	−	9/17 (53%)
Gastrointestinal problems																		
Gastroesophageal reflux	−	+	+	−	−	−	−	+	+	−	−	+	−	−	−	−	−	5/17 (29%)
Constipation	+	−	+	+	+	+	−	−	+	−	−	+	+	−	+	−	−	9/17 (53%)
Diarrhea	−	−	+	−	+	−	−	+	−	−	+	−	+	−	−	−	−	5/17 (29%)
Additional features																		
Increased pain tolerance	+	+	+	+	+	−	+	+	+	+	+	+	+	+	+	+	+	16/17 (94%)
Decreased perspiration/heat intolerance	−	−	+	NK	−	NK	−	NK	+	−	−	NK	−	NK	−	−	−	2/12 (17%)
Recurrent infections	−	+ (otitis, MT)	+ (otitis)	−	−	+ (otitis, upper respiratory tract)	−	+ (otitis, MT)	−	+ (otitis, tonsillitis)	+ (otitis, MT; yeast)	+ (otitis, bronchitis)	+ (otitis, sinusitis)	+ (otitis, MT)	−	−	−	9/17 (53%)
Visual problems	−	+ (strabismus, corrective surgery)	+ (mild hyperopic astigmatism)	−	−	+ (strabismus)	−	−	−	−	−	−	−	−	−	+ (myopia)	+ (myopia)	5/17 (29%)
Congenital heart defect	−	−	−	−	−	−	+ (coronary artery fistula)	−	−	−	−	−	−	−	−	−	−	1/17 (6%)
Renal abnormalities	−	−^c^	−	−	−	−	−^c^	−	−	−	−	−	−^c^	−^c^	−^c^	−^c^	−	0/17
Allergies	+ (penicillin)	+ (food, seasonal)	−	−	+ (food)	+ (penicillin, seasonal)	+ (seasonal)	+ (food)	+ (seasonal)	+ (penicillin)	+ (seasonal)	+ (food, dust, pets)	+ (food)	+ (food)	−	−	−	12/17 (71%)
Asthma	−	−	−	−	−	+	+ (allergy induced)	−	−	−	−	−	−	+	−	−	−	3/17 (18%)
Eczema	−	+	−	−	−	−	−	+	+	+	−	−	+	+	−	−	−	6/17 (35%)
Other							Birth by in vitro fertilization		Sleep apnea			Sleep apnea, atrial fibrillation, intermittent hypoglycemia			Left preauricular skin tag, scoliosis	Episode of idiopathic intracranial hypertension at 12 y		

+ present, − absent, *ASD* autism spectrum disorder, *BDI* Battelle Developmental Inventory, *CSS* Composite Standard Score, *DAS-II* Differential Ability Scales, Second Edition, *DQ* developmental quotient, *EEG* electroencephalography, *F* female, *FSIQ* full scale intelligence quotient, *GCA* general conceptual ability, *ID* intellectual disability, *M* male, *MRI* magnetic resonance imaging, MT myringotomy tubes, *NA* not applicable, *NK* not known, *NV* non-verbal, *NVDQ* non-verbal developmental quotient, *NVIQ* non-verbal intelligence quotient, *PEG* percutaneous endoscopic gastrostomy, *SS* standard score, *VDQ* verbal developmental quotient, *VIQ* verbal intelligence quotient

^a^Individuals not directly evaluated

^b^Monozygotic twins

^c^Normal renal evaluation (ultrasound or computed tomography)

**Table 3:** Dysmorphic features in individuals with *SHANK3* mutations

	S1^a^	S2	S3	S4	S7	S8	S10	S11	S12	S13	S14	Total (%)
Gender	M	M	M	F	F	M	M	F	F	M	M	7 M, 4 F
Age at examination (years)	12	5	7	3	7	9	9	6	42	15	4	3–42
Craniofacial features												
Microcephaly^b^	−	−	−	−	−	−	−	−	−	−	+	1/11 (9%)
Macrocephaly^c^	−	−	−	−	−	−	−	−	+	−	−	1/11 (9%)
Dolichocephaly	−	−	+	−	−	−	−	−	−	−	−	1/11 (9%)
Synophrys	−	−	−	−	−	−	−	−	−	−	−	0
Sparse eyebrows	−	−	−	+	−	−	−	−	−	+	−	2/11 (18%)
Long eyelashes	+	+	−	+	+	+	−	+	+	+	−	8/11 (73%)
Periorbital fullness	+	+	−	+	−	−	−	−	−	+	−	4/11 (36%)
Deep set eyes	−	+	−	+	−	+	+	+	−	−	−	5/11 (45%)
Ptosis	−	−	−	−	−	−	−	−	−	−	−	0
Epicanthal folds	+	+	−	+	−	−	+	−	−	−	+	5/11 (45%)
Hypertelorism	−	−	−	−	−	−	−	−	−	−	−	0
Wide nasal bridge	+	+	−	+	+	+	−	−	−	−	+	6/11 (55%)
Bulbous nose	−	+	−	+	+	+	−	−	−	+	+	6/11 (55%)
Anteverted nares	−	−	−	−	+	−	−	−	−	−	−	1/11 (9%)
Full cheeks	−	−	−	+	−	−	−	−	−	+	−	2/11 (18%)
Malar hypoplasia	+	+	−	+	−	−	−	−	+	+	−	5/11 (45%)
Thin upper vermillion	−	+	+	−	−	−	−	+	−	−	−	3/11 (27%)
Thick lower vermillion	+	+	+	−	−	−	−	−	−	−	−	3/11 (27%)
Short philtrum	−	−	−	−	−	−	−	−	−	−	−	0
Long philtrum	−	+	−	−	−	−	+	−	+	−	−	3/11 (27%)
Malocclusion	+	+	−	+	+	+	+	−	+	−	−	7/11 (64%)
High arched palate	+	+	−	+	−	−	+	−	NE	+	+	6/10 (60%)
Ear anomalies	−	−	−	+ (low set ears)	−	−	+ (overfolded helix)	−	+ (fleshy ears)	−	+ (prominent ears)	4/11 (36%)
Micrognathia	−	−	−	−	−	−	−	−	−	−	−	0
Macrognathia	+	−	−	−	+	−	−	−	+	−	−	3/11 (27%)
Pointed chin	+	−	−	+	+	−	+	+	+	+	−	7/11 (64%)
Hand and feet anomalies												
Large fleshy hands	−	−	−	+	−	−	−	−	−	+	+	3/11 (27%)
5th finger clinodactyly	+	+	−	+	+	+	+	−	+	+	+	9/11 (82%)
Partial syndactyly of toes 2–3	−	+	−	+	−	−	+	−	−	+	+	5/11 (45%)
Sandal gap	+	+	+	NE	+	+	−	NE	−	+	−	6/9 (67%)
Hypoplasia of distal phalanges of 5th finger	−	−	−	−	−	−	−	−	+	+	−	2/11 (18%)
Ectodermal anomalies												
Hypertrichosis	−	−	−	−	−	−	−	−	−	−	−	0
Abnormal hair whorl	−	−	−	−	−	−	−	+	−	+	−	2/11 (18%)
Hypoplastic/dysplastic toenails	+	−	−	+	−	+	−	+	+	−	+	6/11 (55%)
Hypoplastic/dysplastic fingernails	+	+	−	−	−	−	−	−	−	−	−	2/11 (18%)
Other features												
Short stature/delayed growth^d^	+	−	+	−	−	−	−	−	−	+	−	3/11 (27%)
Tall stature/accelerated growth^e^	−	−	−	−	−	−	−	−	−	−	−	0
Hyperextensibility	−	−	−	+	+	+	+	−	NE	+	+	6/10 (60%)
Sacral dimple	−	−	NE	−	−	−	−	−	−	−	−	0
Scoliosis	−	−	−	−	−	−	−	−	−	+	−	1/11 (9%)
Total dysmorphic features	15	16	5	18	10	9	10	6	11	17	11	11/11 (100%)

Only individuals that underwent a detailed evaluation by a clinical geneticist are shown

+ present, − absent, *F* female, *M* male, *NE* not evaluated

^a^S1 also has a de novo pathogenic 17q12 microduplication [62]

^b^Head circumference < 3rd percentile

^c^Head circumference > 98th percentile

^d^Height < 3rd percentile

^e^Height > 98th percentile

##### ASD

Findings of ASD were widespread, with 69% (11/16) receiving a diagnosis of ASD. Among the 11 individuals of the Seaver cohort who received ASD diagnostic testing and a psychiatric evaluation, 82% (9/11) met criteria for ASD on the ADOS and 73% (8/11) met criteria for ASD on the ADI-R. A consensus diagnosis of ASD, accounting for both standardized assessments and clinical impression based on DSM-5 criteria, was reached in 73% (8/11) (Additional file 2: Tables S5, S6). All three children who did not receive an ASD diagnosis (S3, S7, S10) showed relevant features, including two with scores above the ASD cutoff on the ADOS-2 or the ADI-R but not both. It is notable that two of these three individuals (S7 and S10) were verbally fluent with cognitive functioning on the cusp of mild ID/borderline cognitive functioning.

##### Additional behavioral findings

All participants from the Seaver cohort had significant repetitive behaviors (*n* = 14), including hand flapping and stereotypic motor movements (11/14, 79%), chewing and teeth grinding (7/14, 50%), pica and mouthing of objects (8/14, 57%), and stereotypic vocalizations (5/14, 36%). The majority of participants were described as hyperactive (11/17, 65%), although the extent and severity of hyperactivity varied widely as did the extent of impulsivity and inattention. Participants were also prone to aggression (8/17, 47%) and self-injury (3/15, 20%), particularly when frustrated. Sleep disturbance was common (10/17, 59%).

##### Intellectual functioning

ID was observed in all cases that received standardized testing (*n* = 13), with 10 cases falling in the range of a severe-to-profound ID and three cases in the mild range. Two individuals (B2, B3) who did not receive standardized testing were characterized as mildly intellectually disabled based on the extent of language and developmental delay. All individuals within the normed range of up to 68 months (S2, S4, S6, S14) achieved the lowest possible standard score on the Mullen Early Learning Composite (< 49, < 1st percentile), indicating that the instrument reached its lower limit for reliable data collection (“floor” effect). Developmental quotients (DQ) were calculated for all individuals (excluding the 42-year-old individual) and ranged from 6.7 to 30 (mean ± SD, 15.6 ± 8.0). Verbal DQ ranged from 9.2 to 35 (19.9 ± 9.2), and nonverbal DQ ranged from 3.1 to 25.8 (11.39 ± 7.16) (Additional file 2: Table S6). Results from three additional cases who received other cognitive measures (S7, S10, and B1) indicated the presence of mild-to-moderate ID (Table 2).

##### Adaptive behavior

Results from the Vineland-II indicated that adaptive functioning was consistent with cognitive functioning (Additional file 2: Table S5). Overall, motor skills and socialization skills were better developed than communication and daily living skills. Two children (S7, S10) fell within the borderline range; all others fell below the first percentile.

##### Language skills

Language impairment was prominent (17/17, 100%); results are summarized in Table 2. All subjects were delayed in achieving language milestones. With regard to current language abilities, the ADOS-2 (*n* = 11) indicated that five individuals used no words, three used < 5 recognizable words or word approximations, one used mostly single words, and two used complex speech with frequent grammatical errors. Receptive and expressive language were equally delayed (Additional file 2: Table S7). Three individuals (S7, S10, S14) were administered the Peabody Picture Vocabulary Test and achieved scores between < 1st and 7th percentiles. Two of these individuals (S7, S10) achieved scores of 70 (2nd percentile) on the Expressive Vocabulary Test, indicating that despite fluent speech, expressive language abilities were significantly delayed relative to same-aged peers. Two of the Baylor participants were also reported to speak in sentences, but one was mostly echolalic.

##### Motor skills

Most individuals achieved motor milestones on time, despite significant fine and gross motor delays in all participants at the time of evaluation. Hypotonia (16/17, 94%) and gait abnormalities (14/17, 82%) were present in the majority of individuals. Gross motor skills were significantly better developed than fine motor skills (*n* = 9, *p* = 0.02 for both the Mullen and the Vineland-II, Wilcoxon signed-rank test; Additional file 2: Table S7). Two individuals (S7, S10) were administered the Beery Visual-Motor Integration Test and received standard scores of 45 and 65, respectively, which is indicative of visual-motor deficits.

##### Sensory processing

According to parent report, 16 of 17 participants had increased pain tolerance (94%). Results from the Sensory Assessment for Neurodevelopmental Disorders (*n* = 10) and clinical observation indicated that sensory hyporeactivity (i.e., underresponsiveness to stimuli) was prominent. These findings are consistent with the results from the Short Sensory Profile (*n* = 11), indicating high scores in the underresponsive/seeks sensation domain (10/11) and the low energy/weak domain (9/11).

##### Neurological findings

Seizures were reported in five individuals (5/17, 29%), including febrile (*n* = 1), absence (*n* = 3), focal (*n* = 1), and generalized seizures (*n* = 2) (one individual had febrile, absence, and focal seizures) (Table 2). Age of onset ranged from 4 to 14 years (7.2 ± 4). Nine individuals had an abnormal electroencephalography (EEG) (9/17, 53%), including five without clinical seizures. MRI in 15 individuals revealed abnormal findings in five (33%), including white matter abnormalities (*n* = 3), venous angioma (*n* = 1), and mild cerebellar ectopia (*n* = 1).

##### Regression

For the purpose of this manuscript, we only document regression in patients who clearly and consistently acquired skills for a prolonged period of time and then lost these skills, either permanently or for an extended period. Regression, occurring at various stages of development from early childhood to early adolescence, and affecting language, motor, and behavioral domains, was reported in 11 of 17 cases (65%). At least two caregivers noted regression that was triggered by infection and one reported seizures preceding the onset of regression.

##### Other medical conditions

Gastrointestinal problems were common, including gastroesophageal reflux (5/17, 29%), constipation (9/17, 53%), and diarrhea (5/17, 29%). Feeding problems were also common (13/17, 76%), including dysphagia and chewing difficulties; two individuals required placement of a gastrostomy tube. Recurrent infections were reported in 53% (9/17) of individuals, most often affecting the ears. Visual problems, and strabismus in particular, have been described in carriers of 22q13.3 deletions [2, 4, 51] and were present in 29% (5/17) of patients, including strabismus (*n* = 2), myopia (*n* = 2), and astigmatism (*n* = 1). Renal or urinary tract abnormalities, reported in 26–40% of cases with 22q13 deletions [2, 4], were absent in our cohort. Similarly, congenital heart defects, reported in 3–13% of patients with 22q13 deletions [2, 52], were uncommon; one individual had a coronary artery fistula that did not require surgical intervention. Lymphedema, cellulitis, precocious or delayed puberty, hearing problems, and hypothyroidism have been reported in cases with 22q13 deletions [2, 4] but were not present in individuals with *SHANK3* mutations (Table 4).

**Table 4:** Clinical features in individuals with *SHANK3* mutations as compared to 22q13 deletions including *SHANK3*

Clinical features	Individuals with *SHANK3* mutations (current study)	Individuals with 22q13 deletions [2]
Intellectual disability	17/17 (100%)	29/30 (97%)
ASD	11/16 (69%)	26/30 (87%)
Verbally fluent	3/17 (18%)	0/30
Repetitive behaviors	16/17 (94%)	30/30 (100%)
Hyperactivity	11/17 (65%)	14/30 (47%)
Aggression	8/17 (47%)	13/30 (43%)
Sleep disturbance	10/17 (59%)	12/30 (40%)
Hypotonia	16/17 (94%)	23/30 (77%)
Gait abnormalities	14/17 (82%)	13/14 (93%)
Seizures	5/17 (29%)	12/30 (40%)
Abnormal brain MRI	5/15 (33%)	18/26 (69%)
Short stature^a^	5/16 (31%)	3/30 (10%)
Tall stature^b^	0/16	1/30 (3%)
Microcephaly^c^	1/14 (7%)	2/30 (7%)
Macrocephaly^d^	3/14 (21%)	9/30 (30%)
Dolichocephaly	1/11 (9%)	7/30 (23%)
Sparse hair/abnormal whorl	2/11 (18%)	5/30 (17%)
Long eyelashes	8/11 (73%)	13/30 (43%)
Periorbital fullness	4/11 (36%)	8/30 (27%)
Hypertelorism	0/11	3/30 (10%)
Deep set eyes	5/11 (45%)	2/30 (7%)
Ptosis	0/11	2/30 (7%)
Epicanthal folds	5/11 (45%)	9/30 (30%)
Strabismus	2/17 (12%)	3/30 (10%)
Wide nasal bridge	6/11 (55%)	4/30 (13%)
Bulbous nose	6/11 (55%)	15/30 (50%)
Full cheeks	2/11 (18%)	8/30 (27%)
Malar hypoplasia	5/11 (45%)	3/30 (10%)
Long philtrum	3/11 (27%)	5/30 (17%)
Malocclusion	7/11 (64%)	5/30 (17%)
Widely spaced teeth	0/11	1/30 (3%)
High arched palate	6/10 (60%)	8/30 (27%)
Ear anomalies	4/11 (36%)	13/30 (43%)
Pointed chin	7/11 (64%)	7/30 (23%)
Large fleshy hands	3/11 (27%)	17/30 (57%)
5th finger clinodactyly	9/11 (82%)	3/30 (10%)
Syndactyly of toes 2–3	5/11 (45%)	3/30 (10%)
Hypoplastic/dysplastic nails	7/11 (64%)	11/30 (37%)
Hyperextensibility	6/10 (60%)	8/30 (27%)
Scoliosis	1/11 (9%)	7/30 (23%)
Sacral dimple	0/10	4/30 (13%)
Gastroesophageal reflux	5/17 (29%)	13/30 (43%)
Constipation/diarrhea	11/17 (65%)	11/30 (37%)
Increased pain tolerance	16/17 (94%)	26/30 (87%)
Recurrent infections	9/17 (53%)	16/30 (53%)
Renal abnormalities	0/17	12/30 (40%)
Congenital heart defect	1/17 (6%)	1/30 (3%)
Hypothyroidism	0/17	1/30 (3%)
Lymphedema	0/17	7/30 (23%)

*ASD* autism spectrum disorder

^a^Height < 3rd percentile

^b^Height > 98th percentile

^c^Head circumference < 3rd percentile

^d^Head circumference > 98th percentile

##### Dysmorphic features

Dysmorphology examinations were performed on 11 individuals from the Seaver cohort, using a PMS-specific checklist (Table 3, Fig. 1b). All had at least five usually mild dysmorphic features (range 5–18), without a distinctive facial gestalt. In general, findings were consistent with those reported in patients with 22q13 deletions [2, 4–6]. However, some features were more common than previously reported, including fifth finger clinodactyly (9/11, 82%), malocclusion (7/11, 64%), and wide nasal bridge (6/11, 55%) (Table 4). The use of a PMS-specific checklist could account in part for the higher frequency with which certain features were noted. Other features present in over 50% of the individuals were long eyelashes, bulbous nose, high arched palate, pointed chin, hyperextensibility, dysplastic toenails, and sandal gap.

#### Phenotype of individuals with SHANK3 mutations in the literature

The clinical features of 45 previously published individuals with pathogenic or likely pathogenic *SHANK3* variants are summarized in Additional file 1: Table S2. (Fifteen individuals reported only in ClinVar are included in Additional file 1: Table S1 where we summarize the allelic spectrum but are not included here because no phenotype information was available for them.) Although only limited information was available for most cases, the phenotype was consistent with that observed in our cohort, including ID (33/33, 100%), severe language impairment (22/23, 96%), ASD (26/34, 76%), hypotonia (8/12, 67%), seizures (17/30, 57%), and dysmorphic features (13/21, 62%). Regression was reported in 11 individuals.

## Discussion

This is the first study to comprehensively describe the phenotype in patients with PMS due to *SHANK3* point mutations. Our findings demonstrate that loss of *SHANK3* alone is sufficient to produce the characteristic features of PMS, including ID, ASD, severe speech impairment, hypotonia, epilepsy, motor skills deficits, feeding difficulties, mild dysmorphic features, increased pain tolerance, gastrointestinal problems, and neuroimaging abnormalities. In addition, we advance the understanding of the genetic architecture of PMS and, in so doing, provide information to aid in the interpretation of *SHANK3* variants.

### Genetic findings

Findings in our cohort and in previously reported patients indicate that *SHANK3* mutations are fully penetrant. The identification of three families with *SHANK3* mutations in multiple siblings due to germline mosaicism (5%, 3/57) [9, 22, 43] has important implications for genetic counseling. Of note, we identified four recurrent mutations in *SHANK3*, including p.Leu1142Valfs*153, p.Ala1227Glyfs*69, p.Arg1255Leufs*25, and c.2265+1G>A. The most common mutation, c.3679dupG (p.Ala1227Glyfs*69), identified in six individuals, is due to the duplication of a guanine in a stretch of eight guanines, indicating that this segment is prone to replication errors. Functional studies on several of the truncating mutations described here (p.Trp509* in S1, p.Pro834Argfs*59 in S3, p.Lys1670* in S13, and p.A1227Gfs*69 in S7, S8, and B1) provide further support for their deleterious effects [9, 26, 53, 54].

Although the majority of pathogenic/likely pathogenic *SHANK3* variants identified to date are truncating, the interpretation of missense variants remains difficult. Missense variant assessment relies on inheritance, segregation within families, frequency in population databases, functional studies, and computational predictions of pathogenicity (see ACMG guidelines [25]). In the case of *SHANK3*, in silico prediction programs often provide contradictory results (Additional file 1: Table S3). Functional studies could help determine the pathogenicity of missense substitutions; however, previous in vitro analyses have identified synaptic defects associated with missense variants in ASD inherited from healthy parents and found in control databases [9, 53, 54]; hence, more discriminatory functional approaches will need to be developed.

### ASD, ID, language, and motor skills

Our results demonstrate the high prevalence of ASD in individuals with PMS resulting from *SHANK3* mutations, similar to our previous findings in individuals with 22q13 deletions [2]. The ADOS and ADI-R provided important information regarding ASD features, even in individuals with low mental ages; however, clinical evaluation and consensus discussion proved necessary to determine which individuals did not meet criteria for ASD. Negative ASD findings in the two verbally fluent individuals raise questions about the relationship between ASD diagnosis and severe global developmental delay. Interestingly, in spite of severe-to-profound ID, and significant expressive and receptive language delays in the majority of participants, language appears to be more preserved in individuals with *SHANK3* mutations compared to those with 22q13 deletions seen at the same centers [2, 24]. Motor skills deficits were also pronounced, although early motor milestones were achieved on time for the majority of individuals. Gross motor skills were better developed than fine motor skills and, in most cases, appear to be less severely affected than in individuals with 22q13 deletions, particularly regarding gait. These results indicate that *SHANK3* haploinsufficiency affects cognition, language, and motor functioning.

### Regression and psychotic symptoms

Significant cognitive and behavioral regression has been reported in individuals with PMS [2, 3, 9, 51, 55–59]. Over half of our sample reportedly experienced a regression in motor and language skills that occurred during different periods of development (early childhood or adolescence). These results indicate that *SHANK3* haploinsufficiency alone is sufficient to increase risk for regression. However, reports of regression must be interpreted with caution based on a lack of well-defined criteria or standardized assessment instruments and potential recall biases in reporting. Further careful study is needed to characterize the regression phenotype in PMS using longitudinal designs and to begin to elucidate the underlying mechanisms.

Possibly related to regression, psychotic symptoms have emerged as an important area of study in PMS as several reports have suggested that as individuals with PMS age, they may be at increased risk for significant psychiatric disturbance, including bipolar disorder [51, 55–57, 59]. Four of the reported patients had truncating mutations in *SHANK3* [9, 56], indicating that *SHANK3* is responsible for this phenotype. Mutations in *SHANK3* have also been found in four individuals from two families with atypical schizophrenia associated with early onset and ID [22]. The monozygotic twins reported here (B2, B3) showed “manic-like” behavior beginning at 13 years of age in one and at 9–10 years of age in the other. Also, one individual (S12) experienced psychotic symptoms characterized by auditory and visual hallucinations beginning around 12–13 years of age. She had episodic periods of mania and depression, insomnia, decreased appetite and weight loss, unsteady gait, and catatonic posturing, similar to previous reports [51, 55, 56, 59]. Importantly, she also had significant regression in language and motor skills with documented cognitive decline from borderline intellectual functioning before puberty to profound ID based on the current assessment at age 42 (see Table 2). The patient was verbally fluent but became non-verbal. She was also walking independently at 20 months and currently is unable to walk more than several steps without support. Pubertal onset appears to be a potential trigger for shifts in the psychiatric phenotype in PMS; hence, it is important to note that only two of the 14 Seaver participants were post-pubertal.

### Other medical findings

Common medical features in individuals with *SHANK3* mutations were consistent with published literature in subjects with 22q13.3 deletions [1, 2, 4–6]. Epilepsy has been reported in PMS with a mean prevalence of 32% and a wide range of seizure types, frequencies, and severity [24]. The lower frequency of seizures in our study compared to that for previously reported individuals with *SHANK3* point mutations (29% versus 57%) might be due to the young age of many of our patients (seizure onset occurred at ≥ 10 years in 41% [7/17] of new and previously reported individuals). In agreement with our findings, no specific EEG abnormalities have been reported in PMS, and EEG abnormalities (61%) are seen in children with and without a history of clinical seizures [24]. Structural brain abnormalities are observed in about a third of cases with 22q13 deletions (including corpus callosum and cerebellar abnormalities, dysmyelination, ventricular dilatation, and arachnoid cysts) [1, 2, 24]; results from patients with mutations are consistent with those with deletions. Overall, loss of *SHANK3* is sufficient to cause seizures and structural brain changes, although findings remain non-specific to PMS.

Gastrointestinal problems, recurrent infections, and increased pain tolerance were common among individuals with *SHANK3* mutations, consistent with previous estimates in 22q13 deletions [2, 4]. In agreement with these findings, studies in mice showed that SHANK3 is expressed in the spinal cord and primary sensory neurons, where it regulates pain sensitivity [60]. SHANK3 has also been shown to be expressed in intestinal epithelial cells, where it regulates barrier function [61]. In contrast, despite reports of renal and urinary tract abnormalities in 26–40% of cases with 22q13 deletions (including vesicoureteral reflux, hydronephrosis, renal agenesis, and dysplastic or polycystic kidneys) [2, 4], no such anomalies were observed in our cohort. While data from ongoing genotype-phenotype studies are still emerging, it is likely that the genetic risk for renal anomalies is not directly associated to *SHANK3* haploinsufficiency and involves other gene(s) in 22q13.

Despite high variability, mild dysmorphic features were prevalent among patients with *SHANK3* mutations and were consistent with the phenotype in patients with 22q13 deletions [1, 2, 4]. It has been previously reported that the number of dysmorphic features is correlated with deletion size [2] and that several dysmorphic features are associated with larger deletion sizes [7]. Our results suggest that some of the more common dysmorphic features associated with PMS are caused by *SHANK3* mutations, but further studies are needed to determine the contribution of other genes involved in 22q13 deletions.

## Conclusions

This represents a first detailed report of the genetic and phenotypic spectrum associated with *SHANK3* mutations, which are being identified with greater frequency as clinical sequencing becomes more widespread. Our findings show that *SHANK3* haploinsufficiency due to point mutations alone is sufficient to cause a broad range of phenotypic features associated with PMS. These include hypotonia, global developmental delay, ID, ASD, language deficits, sleep disturbance, increased pain tolerance, regression, motor skills deficits, seizures, abnormal EEG, brain imaging abnormalities, feeding difficulties, and gastrointestinal problems. We also describe frequent dysmorphic features in individuals with *SHANK3* mutations, including fifth finger clinodactyly, long eyelashes, bulbous nose, wide nasal bridge, malocclusion, high arched palate, pointed chin, sandal gap, and dysplastic toenails. Importantly, we show that language and motor phenotypes appear to be less severe in individuals with point mutations, as compared to 22q13 deletions. These findings extend the role of *SHANK3* dysfunction in human disease beyond its well-known role at the synapse in the central nervous system.

## Additional files

Additional file 1: **Table S1.** Loss of function and de novo missense variants in *SHANK3* reported previously. **Table S2.** Clinical features of individuals with pathogenic or likely pathogenic *SHANK3* variants reported in the literature. **Table S3.** In silico prediction of pathogenicity of missense variants in *SHANK3* identified in this study and in the literature. **Table S4.** Reported truncating and in-frame variants in *SHANK3* unlikely to be pathogenic. (XLSX 84 kb)

Additional file 2: **Table S5.** Descriptive and diagnostic data by patient. **Table S6.** ASD and intellectual ability classifications in individuals with *SHANK3* mutations. **Table S7.** Language and motor functioning in individuals with *SHANK3* mutations. (PDF 132 kb)

## Abbreviations

ACMG: American College of Medical Genetics and Genomics
ADI-R: Autism Diagnostic Interview-Revised
ADOS-2: Autism Diagnostic Observation Schedule: Second Edition
ASD: Autism spectrum disorder
DAS-II: Differential Ability Scales, Second Edition
DSM-5: Diagnostic and Statistical Manual for Mental Disorders: Fifth Edition
EEG: Electroencephalography
EVS: Exome Variant Server
gnomAD: Genome Aggregation Database
ID: Intellectual disability
IQ: Intellectual quotient
MRI: Magnetic resonance imaging
OMIM: Online Mendelian Inheritance in Man
PMS: Phelan-McDermid syndrome
