# Supplementary material for: Delineation of the genetic and clinical spectrum of Phelan-McDermid syndrome caused by SHANK3 point mutations
Source: Mol Autism. 2018 Apr 27;9:31. doi: 10.1186/s13229-018-0205-9 (PMC5921983; doi:10.1186/s13229-018-0205-9)
Supplement: Supplementary file 2 — Table S5. Descriptive and diagnostic data by patient. Table S6. ASD and intellectual ability classifications in individuals with SHANK3 mutations. Table S7. Language and motor functioning in individuals with SHANK3 mutations. (PDF 132 kb) [file 13229_2018_205_MOESM2_ESM.pdf]

**Table S5.** Descriptive and diagnostic data by patient

| Subject | Age (y) | NVIQ estimate (standard score/DQ) | Vineland Adaptive Behavior Scales-II (standard score) | | | | | ADI-R[a] | | | ADOS-2[b] | | | | | DSM-5 | Consensus diagnosis | Peabody Picture Vocabulary Test-4 (standard score) | Expressive Vocabulary Test-2 (standard score) |
|---|---|---|---|---|---|---|---|---|---|---|---|---|---|---|---|---|---|---|---|
| | | | ABC | Communication | Socialization | Daily Living | Motor | A: Social | B: Comm | C: RRSB | Module | Social Affect | Restricted & Repetitive Behavior | Overall Total | Severity Score | | | | |
| S1 | 12 | 10.4 | 33 | 36 | 38 | 33 | 56 | 23 | 13 | 6 | 1 | 20 | 7 | 27 | 10 | ASD | ASD | ND | ND |
| S2 | 5 | 30.6 | 49 | 44 | 55 | 43 | 61 | 16 | 12 | 3 | 1 | 16 | 3 | 19 | 6 | ASD | ASD | ND | ND |
| S3 | 7 | 18.8 | 49 | 45 | 51 | 52 | 40 | 17 | 10 | 7 | 1 | 2 | 3 | 5 | 2 | No ASD | No ASD | ND | ND |
| S4 | 3 | 19.5 | 55 | 49 | 68 | 55 | 59 | 13 | 12 | 2 | 1 | 19 | 7 | 26 | 10 | ASD | ASD | ND | ND |
| S6 | 5 | 35 | 53 | 54 | 49 | 53 | 64 | 25 | 21 | 6 | 1 | 15 | 5 | 20 | 8 | ASD | ASD | ND | ND |
| S7 | 7 | 74 | 75 | 75 | 87 | 69 | 75 | 12 | 11 | 1 | 3 | 12 | 3 | 15 | 9 | No ASD | No ASD | 75 | 70 |
| S8 | 9 | 14.6 | 53 | 53 | 51 | 54 | 59 | 28 | 14 | 6 | 1 | 20 | 8 | 28 | 10 | ASD | ASD | ND | ND |
| S10 | 9 | 60 | 72 | 72 | 78 | 71 | 88 | 6 | 4 | 3 | 3 | 5 | 1 | 6 | 3 | No ASD | No ASD | 78 | 70 |
| S11 | 6 | 17.3 | 42 | 36 | 53 | 34 | 51 | 28 | 14 | 7 | 1 | 18 | 2 | 20 | 7 | ASD | ASD | ND | ND |
| S12 | 42 | 0.97 | ND | ND | ND | ND | ND | ND | ND | ND | ND | ND | ND | ND | ND | ND | ND | ND | ND |
| S13 | 15 | 15.5 | 36 | 37 | 43 | 35 | 59 | 29 | 13 | 4 | 1 | 14 | 5 | 19 | 6 | ASD | ASD | ND | ND |
| S14 | 4 | 33 | 49 | 42 | 61 | 46 | 56 | 21 | 13 | 5 | 1 | 18 | 2 | 20 | 7 | ASD | ASD | 60 | ND |

[a] ADI-R cutoff scores for autism are: Social = 10, Communication (nonverbal) = 7, Repetitive behaviors and restricted interests = 3.

[b] ADOS-2 Module 1 (Pre-verbal/single words) cutoff scores for autism and ASD are 16 and 11 for *few to no words,* and 12 and 8 for *some words*. ADOS-2 Module 3 (Fluent speech) cutoff scores for autism and ASD are 9 and 7, respectively.

*ABC* Adaptive Behavior Composite, *ADI-R* Autism Diagnostic Interview-Revised, *A: Social* Qualitative abnormalities in reciprocal social interaction, *B: Comm* Communication, *C: RRSB* Restricted, repetitive, or stereotyped patterns of behavior, *ADOS-2* Autism Diagnostic Observation Schedule, Second edition, *DQ* developmental quotient, *DSM-5* Diagnostic and Statistical Manual of Mental Disorders, Fifth edition, *IQ* intellectual quotient, *NVIQ* nonverbal IQ, *ND* not done, *y* years

**Table S6.** ASD and intellectual ability classifications in individuals with *SHANK3* mutations

| Classification | n | % |
|---|---|---|
| ASD classification (n=11) | | |
| ASD classification on ADOS | 9 | 82 |
| ASD classification on ADI-R | 8 | 73 |
| Consensus diagnosis of ASD | 8 | 73 |
| Nonverbal IQ classification (n=12) | | |
| Mild ID (IQ 55-69) | 2 | 17 |
| Severe ID (IQ 25-40) | 3 | 25 |
| Profound ID (IQ <25) | 7 | 58 |

*ASD* autism spectrum disorder, *ADI-R* Autism Diagnostic Interview-Revised, *ADOS-2* Autism Diagnostic Observation Schedule, Second Edition, *ID* intellectual disability, *IQ* intellectual quotient

**Table S7.** Language and motor functioning in individuals with *SHANK3* mutations

| Domain | Mean age equivalent ± SD in months (range) |
|---|---|
| **Language** | |
| Receptive language | |
| Mullen | 10.0 ± 3.1 (5-14) |
| Vineland-II | 12.6 ± 5.3 (6-21) |
| Expressive language | |
| Mullen | 7.9 ± 6.0 (2-23) |
| Vineland-II | 10.2 ± 4.2 (7-21) |
| **Motor** | |
| Fine motor skills | |
| Mullen | 16.7 ± 5.3 (9-27) |
| Vineland-II | 20.4 ± 10.5 (6-36) |
| Gross motor skills | |
| Mullen | 22.2 ± 4.7 (16-28) |
| Vineland-II | 26.8 ± 6.1 (17-36) |

Language and motor age equivalents in individuals assessed with both the Mullen Scales of Early Learning and the Vineland Adaptive Behavior Scales II (n=9; S1-S4, S6, S8, S11, S13, and S14).
